# Experimentally simulating quantum walks with self-collimated light

**DOI:** 10.1038/srep28610

**Published:** 2016-06-29

**Authors:** F. Qi, Y. F. Wang, Q. Y. Ma, W. H. Zheng

**Affiliations:** 1 State Key Laboratory on Integrated Optoelectronics, Institute of Semiconductors, CAS, No. 35A, Qinghua East Road, Haidian District, Beijing, Post Code: 100083, China; 2 Laboratory of Solid State Optoelectronics Information Technology, Institute of Semiconductors, CAS, No. 35A, Qinghua East Road, Haidian District, Beijing, Post Code: 100083, China; 3 College of Materials Science and Opto-Electronic Technology, University of Chinese Academy of Sciences, No. 19A Yuquan Road, Beijing, Post Code: 100049, China

## Abstract

In self-collimated photonic crystal, periodically arranged air holes of sub-wavelength scale provide flattened equi-frequency curves perpendicular to the ΓM direction, which allow light or photons propagating in a quasi-uniform medium without diffraction. Here we for the first time experimentally simulate four-step single-photon discrete time quantum walks with classical light in such a photonic crystal chip fabricated on silicon-on-insulator. Similarities between theoretical expectations and experimental results are higher than 0.98. The functional area is compact and can be extended to construct more complicated linear quantum circuits.

Quantum walks (QWs) have emerged as a surprising evolution process in computer science and quantum physics since their potential in quantum algorithms^1^, universal quantum computers^2^ and quantum simulations^3^,^4^,^5^. These schemes are promising for their unprecedented calculation acceleration and thus can solve problems that are hard for classical computers^6^. The wave aspect of the particle is highly revealed in QWs^7^. Photon, an evident combination of wave and particle, has been demonstrated as an excellent “walker” since they are easy to generate and manipulate. QWs of photons have been realized in several integrated optical platforms such as silicon oxynitride^8^, borosilicate glass^4^,^5^, and silicon-on-insulator (SOI)^9^. Evanescently coupled parallel waveguides array can be used to perform continuous-time QWs, and discrete beam splitters array can be used to perform discrete-time QWs. Further development in QWs requires large scale integration and manipulating photon propagations in more flexible ways^9^,^10^,^11^,^12^,^13^. On the other hand, series of quantum phenomena, including single-photon QWs, can be simulated by classical coherent waves^14^,^15^,^16^,^17^. The reason is that the single photon and many photon problems are described by the same probability distributions. In particular, for waveguide arrays, the paraxial evolution equation of optical beams can resemble the Schrodinger equation^14^,^17^. Except for QWs, Anderson localization^18^,^19^, Bloch oscillations^18^,^20^, topological phases^21^,^12^ and parity-time symmetry^17^,^22^ have also been simulated with classical coherent light.

Diffraction is a fundamental process in wave optics. Optical beams will diffuse in space as they propagate. Photonic crystals (PCs) can be engineered to generate highly anisotropic 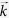
(wave vector) space equi-frequency contours (multiple curves with equal frequencies) with flat sections, thus prohibit spatial diffusion even without waveguide structure. Centimeter-scale non-diffraction propagation in PCs has been achieved on SOI^23^. This phenomena, or self-collimation of PCs^24^, can be useful in several ways. The simplest functional units are the beam splitter and interferometer based on line-defect splitters and slot reflection mirrors^25^, which can be used in photonic circuits^26^,^27^,^28^ and photonic sensors^29^. Theoretically, there is no crosstalk when two perpendicular self-collimated beams intersect, which will simplify the circuits design. The self-collimated PCs fabricated on SOI are also compact and with low loss^23^, thus are promising for ultra-compact and large-scale integration. However, experimental results are limited to very simple circuits so far, such as long distance self-collimated propagation^23^ and Mach–Zehnder interferometers^28^. The main obstacles include experimental realization of beam splitters, and circuit layout designs for specific applications.

Here, we present an experimental simulation of QWs with classical coherent light in a self-collimated PC chip fabricated on SOI, where light propagates in a self-collimated way. Specific layout design is carried out to ensure a four-level cascading beam splitter array working with minimal crosstalk. Then four-step Hadamard single-photon QWs are simulated in the near-infrared band^8^,^16^.

## Results

### Design of the self-collimated beam splitter

**Figure 1:**
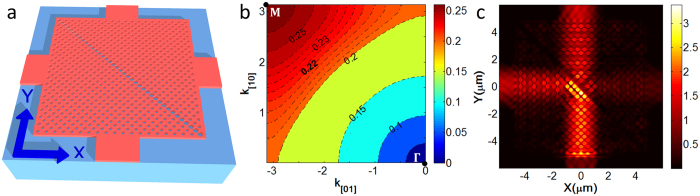
Design of the self-collimated PC beam splitter. (**a**) self-collimated beam splitter. The splitter is constructed via a line defect with enlarged air-hole radius. The whole square lattice has been rotated by 45°. (**b**) equi-frequency contour of the lowest TE-like band. (**c**) 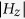 field of the 1:1 beam splitter at 1560 nm, calculated by 3-dimensional FDTD method.

The PC is formed by etching square-lattice air holes into the top silicon layer (220 nm in thickness) of the SOI. The equi-frequency contour of the lowest TE-like band is calculated by 3-dimension plane wave expansion method, and the result is presented in Fig. 1b. In the calculation, the slab thickness is 0.627*a* and air-hole radius is 0.31*a*, where *a* is the lattice constant. Flattened equi-frequency curves can be found within the normalized frequency (*a*/*λ*) range 0.22~0.23. The group velocity, defined by 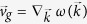, is perpendicular to the equi-frequency curves^30^, i.e. along ΓM direction in Fig. 1b. In real space, ΓM direction is parallel with the diagonal of the square lattice. Then, the 3-dimensional finite difference time domain (FDTD) method is used to determine that a lattice constant *a* = 351 nm is optimal for self-collimated propagation at a wavelength of 1560 nm. The self-collimated propagation can be achieved in a broad spectral band, as we can see that the equi-frequency curves are nearly flat within a frequency range. As shown in Fig. 1a, the self-collimated beam can be split by a 45° reflector, which is a line defect with enlarged air-hole radius that splits the beam of 1560 nm equally when the air-hole radius is 156 nm. The steady-state magnetic field of the splitter is presented in Fig. 1c.

### Circuit design and quantum mechanical model

**Figure 2:**
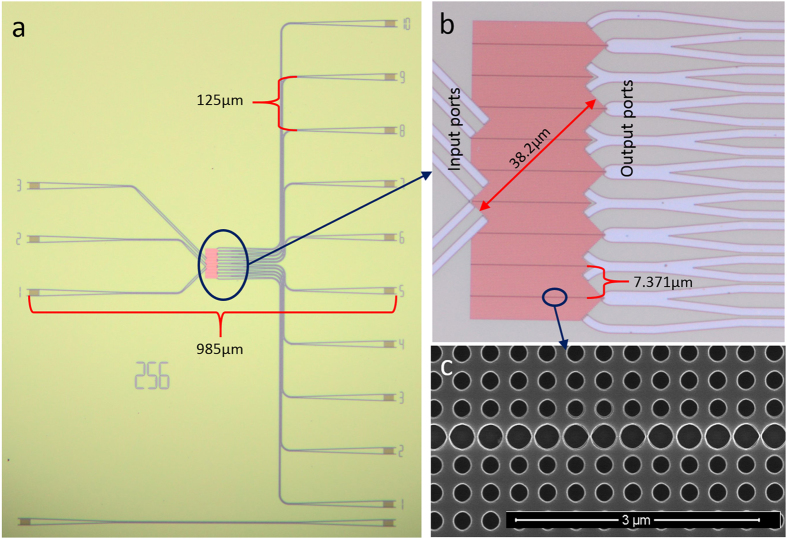
Circuits layout. (**a**) optical microscope picture of the whole circuit. The three ports on the left are input ports and the ten ports on the right are output ports. (**b**) PC region of the circuit, in which light will experience four reflections or transmissions perform. (**c**) Scanning electron microscope image of the splitter.

The layout of the beam splitter array combined with external input and output ports are shown in Fig. 2a,b. The PC structure has been rotated by 45° with respect to that in Fig. 1a. As a result, the input and output waveguides can be guided to the same direction by 45° curved waveguides. This layout design can avoid the intersection of the output waveguide and match most waveguide chip testing systems. Three strip waveguides with a width of 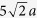 are used to couple light into the splitter array, and ten strip curved waveguides with a width of 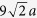 are used to extract light from the splitter array. Here the output waveguides are broadened to compensate the beam shift caused by Goos-Hänchen displacement. At the output ports, all the strip waveguides are tapered and bent in the same manner, which ensures that the tapering and bending losses at each of the output ports are the same.

**Figure 3:**
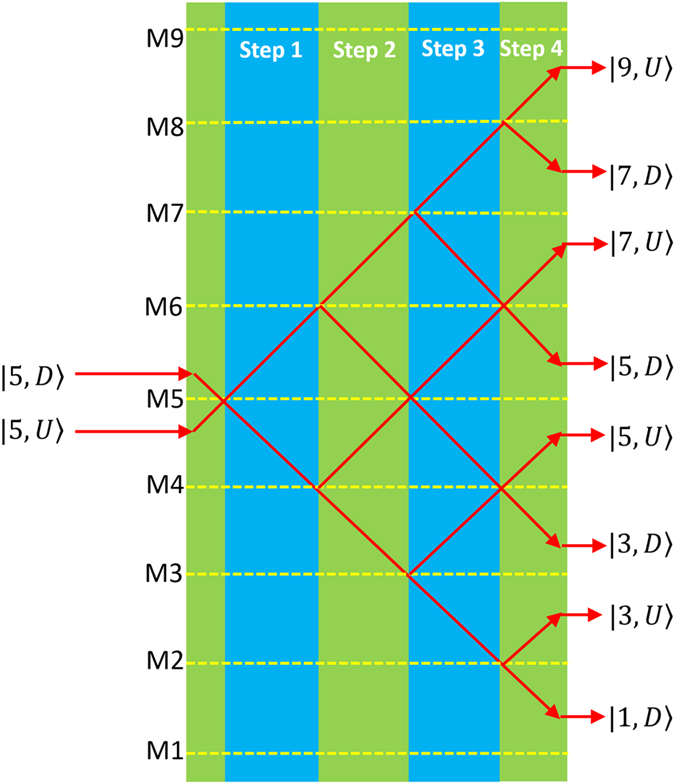
Schematic diagram of the four-step discrete time QWs. The splitter is labeled by M*n* (*n* = 1, 2, …, 9), and the state vectors of the walker combined with the “coin” freedom are 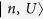 and 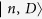

In the PC region, nine parallel line defects are equally spaced by 21*a*, as shown in Fig. 2b. Once injected into this region, each beam will experience four reflections or transmissions. This circuit can perform a four-step discrete time QWs. The walking process is illustrated in Fig. 3. The walker is a photon with its position freedom identified by the splitter number. The “coin” space is represented by the propagating direction, 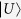 for upwards and 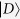 for downwards. One walk step is as follows: a walker in 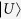 or 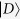 state reaches the splitter M*n*, then the “coin” space is transformed by the splitter according to


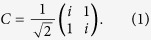


After that the evolution operator 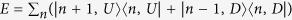
is implemented on the walker. For example, if the initial state is 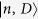, the final state after four-step walks is


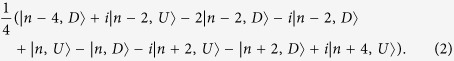


Here, the “coin” operator is equivalent to the commonly used Hadmard gate, and the process is four-step Hadmard QWs.

### Experiment

**Figure 4:**
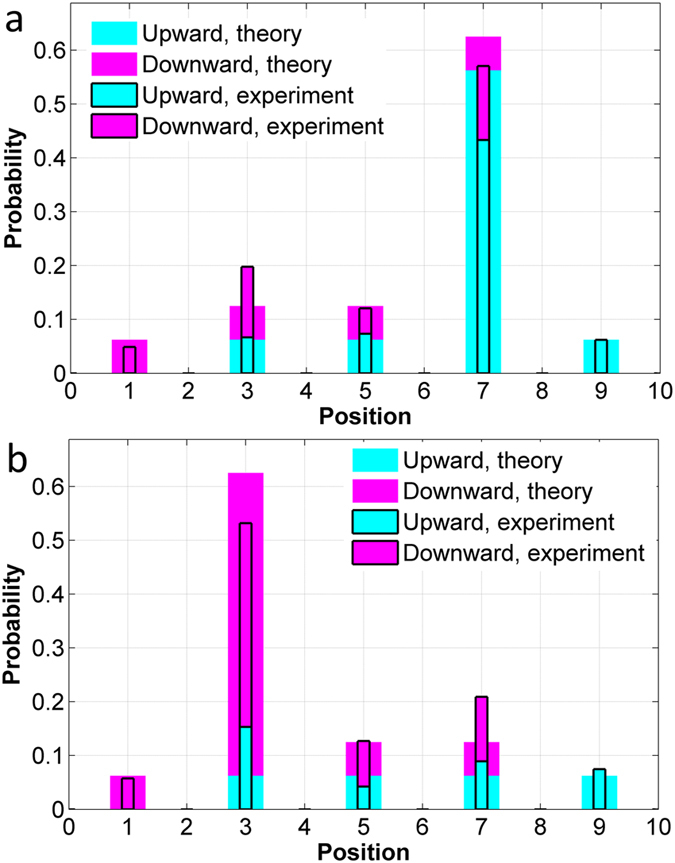
Experimental results. (**a**) upwards initial state. (**b**) downwards initial state. The position coordinate should be referred to Fig. 3, not those in Fig. 2a.

The circuit is fabricated by electron beam lithography (JOEL JBX6300FS) and inductively coupled plasma etching. Grating couplers are used to couple TE polarized light in and out of the chip. A classical coherent source centered at 1560 nm is coupled into one of the input ports to simulate single-photon QWs. An optical spectrometer is used to collect the powers at the output ports, and the results are presented in Fig. 4. Both sets of the powers are normalized to 1, thus can be treated as probability distributions. The upwards and downwards initial states are simulated by coupling light into port 1 and port 3 (Fig. 2a), respectively.

Measured distributions are compared with the theoretical ones by calculating the similarity 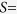 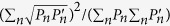, where *n* represents the position. The results are *S* = 0.9852 for the upwards initial state, and *S* = 0.9849 for the downwards initial state. We can also calculate the similarity associated with the “coin” state, which is expressed by 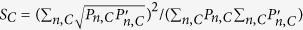, where *C* = *U*, *D*. The results are *S*_*C*_ = 0.9543 for the upwards initial state, and *S*_*C*_ = 0.9467 for the downwards initial state. The degradation in similarity mainly comes from the splitting ratio deviating from 1:1. The reflections at the intersection of the PC and strip waveguides also contribute to the degradation.

Finally, the average loss of the circuit within a 40 nm band (1540 nm~1580 nm) is estimated with a series of structures fabricated on the same chip (see Supplementary Fig. S1). The propagation loss of the self-collimated beam is 0.094 dB/μm, and the insertion loss of the line defect splitter is 0.806  dB. The loss (reflection loss and scattering loss) induced by a pair of intersections between the PC and strip waveguide is 1.11 dB. With this in hand we can estimate that the loss in the PC region of the circuits is 7.92 dB. However, the strip waveguides of the output ports are simply broadened to compensate the Goos-Hänchen displacement, which will also reduce the scattering loss. Finally, the directly measured total loss in the PC region is 6.51 dB. For further study, the propagation loss can be reduced by improving the lithography and dry etch process^23^. The insertion loss can be improved by using slot-based splitter^31^, but the obstacle to application is the fabrication of the air slot.

## Discussions

Benefitting from the highly anisotropic spatial dispersion, light propagates in a self-collimated way. Compared with another discrete-time QWs circuit also fabricated on SOI^9^, the functional area (PC region) is compact and only has a footprint of 30.9 μm × 69.5 μm. It can be further reduced by decreasing the separation between splitters. The only restriction is the beam width of the self-collimated propagation. Compared with the surface plasmonic quantum circuits^32^,^33^, the footprint of the self-collimated PC is larger but the propagation loss is considerably reduced. On the other hand, it is straightforward to control the reflectivity of the self-collimated beam splitters^26^,^31^, which can be further used as unitary operators in quantum circuits^34^. Thus the self-collimated PCs can be developed to construct more compact linear optical quantum networks, which is essential to quantum computers^35^.

In conclusion, we have demonstrated a self-collimated PC chip for discrete-time QWs. Experimental simulation of single-photon QWs is conducted with classical coherent light, and the similarities are as high as 0.98. The self-collimated PC platform proposed here is promising for future silicon-based ultra-compact quantum circuit.

## Additional Information

**How to cite this article**: Qi, F. *et al.* Experimentally simulating quantum walks with self-collimated light. *Sci. Rep.* **6**, 28610; doi: 10.1038/srep28610 (2016).

## Supplementary Material

Supplementary Information
