# Supplementary material for: Experimentally simulating quantum walks with self-collimated light
Source: Sci Rep. 2016 Jun 29;6:28610. doi: 10.1038/srep28610 (PMC4926089; doi:10.1038/srep28610)
Supplement: Supplementary Information [file srep28610-s1.pdf]

Supplementary materials for

Experimentally simulating quantum walks with self-collimated light

F. Qi[1, 2, 3*], Y.F. Wang[2*], Q. Y. Ma[1, 2, 3], and W.H. Zheng[1, 2]

[1]State Key Laboratory on Integrated Optoelectronics Lab, Institute of Semiconductors, CAS.
[2]Laboratory of Solid State Optoelectronics Information Technology, Institute of Semiconductors, CAS.
[3]College of Materials Science and Opto-Electronic Technology, University of Chinese Academy of Sciences.

1. Characteristics of the self-collimated propagation and beam splitter

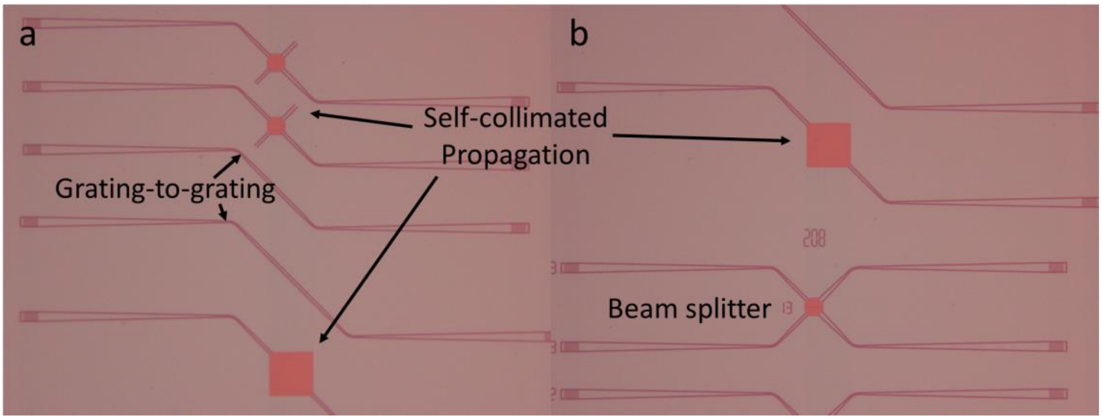

Fig S1| Characteristics of self-collimated propagation and 1:1 beam splitter. a and **b**, the structure used to estimate the loss.

We estimate the average loss through the structure presented in Fig. S1. An amplified spontaneous emission source together with the optical spectrometer are used here. The average splitting ratio (R/T) within the 40 nm band (1540 nm~1580 nm) is 0.95 and 0.91. The lengths of the self-collimated propagation are $185\sqrt{2}a$ and $60\sqrt{2}a$ (a = 351 nm), respectively. The structure in the middle of Fig S1a, where two gratings are connected with tapered, bended and straight strip waveguides, is used to measure the grating-to-grating spectrum.

2. Reflection of the grating back into strip waveguide

Fully-etched one-dimension grating coupler is used here to simplify the fabrication process, but the reflection of the grating back into the strip waveguide is high. Thus the Fabry–Pérot (FP) interference is very strong. The FP oscillation period is about 1 nm in the spectra of the beam splitter, and about 0.6 nm in the QWs circuit present in the main text, due to longer propagation distance (within the waveguide and the PC region). This limits the minimum bandwidth that can be used in the experiment. In the experiment, the bandwidth of the coherent source is 1 nm.

The standard shallow-etched grating couplers can significantly improve this, but need a two-step lithography[1]. Another feasible way is end-fire coupling with inverse taper[2,3], which also need extra lithography steps.

3. Reflection at the waveguide-PC interface

There also exists a reflection of 7.3% at the intersection between the PC and external strip waveguide in FDTD calculation. As a result we can find back reflection in the negative X direction in Fig. 1c. The reflection at two intersection also lead to FP interference, thus affect the performance of the device when involved with narrowband laser light or photons. In experiment this reflection is somewhat reduced because of the slightly tilted air-hole sidewalls. However the scattering loss of the intersection is obvious, thus we can measure a total loss induced by a pair of intersections as high as 1.11dB. The oscillation period is about 10 nm in the spectrum of the beam splitter presented in Fig. S1, and about 6 nm in the spectrum of the QWs circuit. Nevertheless, the reflections in experiment are small. The effects on the similarities are small even though the bandwidth of the injecting wavelength is much smaller than 6 nm.

However, the bandwidth of single photons generated by type-I or type-0 spontaneous parametric down-conversion[4,5], as well as periodically poled lithium niobate (Aurea TPS_1550)[6,7], can be tens of nanometers to one hundred. Generally, spectral filtering are needed before photons are coupled into chips, and the majority of the energy is lost. Considering the broadband properties of the self-collimation, QWs with broadband photons are possible with the chip introduced here.

References

1. Taillaert, D. *et al.* Grating couplers for coupling between optical fibers and nanophotonic waveguides. *Jpn. J. Appl. Phys.* **45,** 6071 (2006).
2. Almeida, V. R., Panepucci, R. R. & Lipson, M. Nanotaper for compact mode conversion. *Opt. Lett.* **28,** 1302–1304 (2003).
3. Xu, X. *et al.* Near-infrared Hong-Ou-Mandel interference on a silicon quantum photonic chip. *Opt. Express* **21,** 5014–5024 (2013).

4. Fiorentino, M. *et al.* Spontaneous parametric down-conversion in periodically poled KTP waveguides and bulk crystals. *Opt. Express* **15,** 7479 (2007).
5. Baek, S.-Y. & Kim, Y.-H. Spectral properties of entangled photons generated via type-I frequency-nondegenerate spontaneous parametric down-conversion. *Phys. Rev. A* **80,** 033814 (2009).
6. Jin, H. *et al.* On-Chip Generation and Manipulation of Entangled Photons Based on Reconfigurable Lithium-Niobate Waveguide Circuits. *Phys. Rev. Lett.* **113,** (2014).
7. Home - Auréa Technology - photon counting solution. Available at: http://aureatechnology.net/en/. (Accessed: 17th April 2016)
